# Medical Voluntarism and Orthopaedic Advancements: Lancashire and the Disabled Ex-Servicemen of the First World War

**DOI:** 10.1093/shm/hkae029

**Published:** 2024-06-12

**Authors:** Nicola Smith

**Affiliations:** Department of History, Politics and Philosophy, Manchester Metropolitan University, Manchester M15 6LL, UK

**Keywords:** medical voluntarism, Grangethorpe Hospital, orthopaedics, rehabilitation, disabled ex-servicemen

## Abstract

This article explores the fundamental role of Lancashire’s medical voluntarism in providing restorative orthopaedic treatments to the region’s First World War, disabled ex-servicemen and assisting in their return to society. It offers a case study of orthopaedic treatments and schemes of rehabilitation provided at Grangethorpe Hospital, Rusholme, between 1914 and 1918. Forming a regional comparison to existing histories of First World War disabled ex-servicemen, which focus primarily on the interwar period, this article traces continuities in pioneering medicine and examples of Lancashire-based medical individuals and institutions. In doing so, this article demonstrates how the region’s response to disablement during the Industrial Revolution underpinned the construction of charities and the advancement of orthopaedic treatments required to provide rehabilitative care during the First World War. Moreover, this paper situates Lancashire and its commitment to medical voluntarism and the reconstruction of disabled ex-servicemen as a key site in the UK’s history of voluntarism.

Voluntary action during the First World War was more significant than during any other British history period. While over two and a half million men volunteered to fight, a similar number of British citizens volunteered to support the war effort at home, with 18,000 new charities created during the war.^1^ While the act of non-uniformed volunteerism and philanthropic efforts at home during 1914 and 1918 may not have been as dangerous as those in conflict, the scale of voluntary contribution during this period was unprecedented but remains an under-researched history.

Recent scholars, including Emily Bartlett and Hugh Cunningham, have explored the contributions and reputation of philanthropy and charitable care provided to disabled ex-servicemen of the First World War. Bartlett considers shifting ideas of disability, work and gender through charitable employment schemes available to disabled ex-servicemen during the interwar period.^2^ Furthermore, Cunningham and Bartlett explore how voluntary approaches to rehabilitation and employment shaped popular responses to disabled ex-servicemen. Moreover, Deborah Cohen’s research is one of only a few studies to consider the significance of the relationship between state and voluntary support for disabled veterans of the First World War.^3^ Highlighting the importance of voluntary efforts in Britain’s interwar stability, Cohen argues that the state’s disregard left philanthropists to construct a social peace amongst a public eager to provide the gratitude of an uninterested state.^4^

Providing an alternative to accounts of interwar voluntarism, Meaghan Kowalsky suggests that while the First World War highlighted the inadequacies in pre-existing disablement welfare, those who cared for disabled ex-servicemen between 1914 and 1918 did so in an enlightened manner than had been previously contended.^5^ This article, therefore, extends this analysis to focus on regional advancements in the provisions of medical voluntarism and orthopaedic care accessible to disabled ex-servicemen within Lancashire between 1914 and 1918, strengthened by the region’s social awareness of the barriers faced by disabled people.

## Background

As the First World War progressed, the severity of mutilation upon the bodies of returning men was unprecedented, and medical services both nationally and within Lancashire were unprepared for the physical devastation.^6^ Despite Lancashire’s medical heritage and experience in treating the physical disabilities of the Industrial Revolution, the scale of orthopaedic injuries arriving during the First World War required an enlightened approach to the elementary medical treatments available. While the arrangements for general medical care and the region’s hospital facilities expanded to sustain the capacity of an increasing number of returning men, the shortage of orthopaedic beds and aftercare facilities challenged Lancashire’s medical men, with one-quarter of the region’s patients suffering an orthopaedic injury.

However, voluntary contribution was the archetypal response to the social welfare inadequacies of the working classes of Lancashire during the eighteenth and nineteenth centuries and is demonstrative of the cultural movement of philanthropy exhibited within the region and their response. Therefore, examining the evolution of medical voluntarism within the industrial district of Lancashire has many advantages. It provides an alternative to other histories of philanthropy and charity that overlook the significance of working-class agency and the influence of local political and cultural developments that shape the foundations and purpose of its charities.

The evidence of this article demonstrates how the region embraced a culture of care attitude towards the welfare provisions required to support disability and established a voluntary care system amongst community members suffering from physical impairments. While often overlooked for post-war medical and social advancements, it contends that the First World War period (1914 and 1918) was an instrumental moment in military medicine and a turning point in the history of disability. Through an examination of the Manchester Medical Collection Section 3-16 and regional clinical literature, this evidence offers an example of an emerging network of pioneering orthopaedic surgeons advocating for the formation of specialist centres and rehabilitative treatments. Moreover, Lancashire offers a persuasive example of the clinical developments of orthopaedic practice that established a national blueprint of surgical and rehabilitative treatments, modernising the practice and underpinning present-day orthopaedics.

## An Orthopaedic Response

The physical destruction of modern warfare between 1914 and 1918 mutilated and wreaked havoc on men’s bodies more devastatingly than previous conflicts due to the increased effectiveness of artillery and the medical complications arising from uncleanliness and the absence of antibiotics in this period.^7^ Over 1 million British ex-servicemen returned home from the First World War with permanent disabilities, and during the first year of conflict, the medical resources and military surgeons at the front and home were quickly overwhelmed by the scale of dismemberment with an unprecedented number of amputations performed.

One-quarter of casualties arriving into British military hospitals sustained orthopaedic injuries, with over 41,000 limbs amputated between 1914 and 1918 and a further 272,000 suffering injuries to limbs that did not require amputation.^8^ These figures revealed the level of mutilation experienced by men throughout the First World War, endorsing the requests from orthopaedic surgeons to implement new and advanced special medical centres focussed on treating fractures, nerve injuries and artificial limb fitting.^9^ Additionally, the increasing sight of returning dismembered men reinforced the cause, with surgeons outlining the painful and unfulfilling quality of life a permanently disabled serviceman would lead as a civilian without further medical care and rehabilitation.

The arrival of returning disabled ex-servicemen to England and Wales continued to increase rapidly after the outbreak of war. By February 1915, an average of 360 disabled men were returning home each month. Lancashire, in particular, experienced unprecedented numbers of orthopaedic injuries requiring long-term treatment and rehabilitation. Furthermore, by Spring 1915, Manchester experienced the highest number of returning disabled ex-servicemen outside London, with over 9,000 men passing through the town. A large proportion of patients sent to Manchester had experienced shrapnel wounds involving large fragments of casing, bullet wounds and frostbite. These disabled ex-servicemen received medical and rehabilitative treatments, and the daily appearance of groups of unemployed, disabled ex-servicemen on crutches with pinned trousers and jackets covering missing limbs became an increasingly common presence.^10^

Details in the region’s hospital medical case notes demonstrate that these surgical cases required lengthy hospitalisation and rehabilitation due to limb and subsequent nerve damage. Encouragingly, several leading orthopaedic and neurological surgeons worked within Lancashire with the foresight to utilise the region’s experimental character to advocate for the expansion of innovative medicine and technologies. Lancashire had already established its first special orthopaedic centre at Alder Hey Hospital, Liverpool, in 1915, for reconstructive surgery led by the distinguished orthopaedic surgeon and Director of Military Orthopaedics in Britain Robert Jones (1857–1933).^11^

Robert Jones was a distinguished orthopaedic surgeon whose reputation and experience in treating fractures and supporting those with permanent disabilities assisted him in securing influence and the necessary resources to plan his vision of an orthopaedic practice to treat disabled men during the First World War.^12^ Jones’s medical career began at an early age under the practice of his uncle, Hugh Owen Thomas, considered the father of orthopaedics.^13^ While not credited as a trained surgeon, Thomas’s medical splints, notably the Thomas Splint, assisted with the healing of bone fractures, which contributed significantly to orthopaedic treatments, especially during the First World War.

Introduced to the front by Robert Jones in 1916, the Thomas Splint was initially designed to quickly stabilise the femur bone and reduce the loss of excessive blood and handling of the wound, which allowed the patient to arrive at the Casualty Clearing Station in a healthier condition with a reduced level of shock and risk of infection. The use of the Thomas Splint during the First World War considerably lowered mortality from femur fractures from 80 to 20 per cent.^14^ Jones gained much inspiration from his uncle’s expertise and continued implementing and modifying orthopaedic splints as post-war routine clinical treatments. This experience and distinctive practice under Thomas proved invaluable during Jones’s appointment as Surgeon Superintendent to the construction of the Manchester Ship Canal in 1886, where he formed one of the world’s first accident casualty services.

The construction of the Manchester Ship Canal between 1889 and 1895 enabled vessels of up to 18,000 tons to sail to Manchester from the Mersey estuary through Runcorn and Warrington, bypassing the port of Liverpool.^15^ A navvy’s occupation was considered amongst the most hazardous of manual jobs due to the continuous risk of severe injury and accidental death; however, the medical care available to them remained inadequate and the responsibility of voluntary and workhouse infirmaries. Throughout the 6-year construction of the canal, Jones treated over 3,000 fracture cases and operated on over 300 of the most severe injuries, including amputations and artificial limb fittings.^16^ During this time, he formed an understanding and admiration for the hardworking but socially and medically disadvantaged navvies who taught him the importance of applying a quick and effective medical service through good communication to ensure the functional efficiency of the workforce.^17^ By supervising the unpredictability of daily working activities and primitive conditions of the navvies, Jones would coalesce his pre-war experiences and willingness to apply experimental treatments to form a similar care system for disabled ex-servicemen during the First World War. Jones’s interest and expertise in orthopaedic practice progressed productively before the outbreak of war in 1914, and he continued to influence the modernisation of the field through posts held as honorary surgeon to the Royal Southern Hospital and his private clinic both in Liverpool.

However, it was during the early twentieth century that Jones’s expertise in observation and providing disabled people with an effective service through surgery and therapy that relieved pain and provided comfort. Much of this experience was developed during his time as Honorary Surgeon at the Baschurch Convalescent Home for Children, the first fully open-air hospital.^18^ Here, Jones worked with the hospital founders and trained nurses Agnes Hunt and Emily Goodford, with monthly visits to the Home’s disabled children, providing a medical examination of the disabilities and congenital deformities.^19^

At the Baschurch Home, Jones mastered the advantages of applying therapies to permanent disabilities at the earliest possibility through gentle but effective treatments that often included the use of plasters, frames and splints in an observed and continued care plan.^20^ Jones’s work at Baschurch advanced his understanding of orthopaedics and treatments offered in the private and voluntary homes, which, together with Agnes Hunt, inspired the transformation of Baschurch into the first country orthopaedic hospital. Much of Jones’s time spent working with disabled children and navvies before the war influenced his foresight to change the clinical and moral approach to orthopaedic and long-term aftercare that would reinforce the reconstruction scheme available to the disabled ex-servicemen of the First World, which contributed considerably to modern-day orthopaedics.^21^

Jones’ willingness to include experimental and controversial medicine, including ‘open-air’ treatments in half-open huts, provides an additional example of Jones’s ambition to improve the quality of treatment and aftercare for patients with orthopaedic injuries. Like other new medical provisions, the huts faced opposition, described by Hunt in her memoirs, as inciting a ‘medical opinion that was doubtful of the cold, so consent was given rather anxiously’.^22^ Despite opposition to open-air treatments, Jones considered the effects of fresh air and sunshine to act antiseptically upon the body, reducing infection and mortality. Jones’s time and influence at the Home are reflected in the advancements of its medical facilities and innovative technology, especially its recognition as the first British medical institution to introduce the use of X-ray imagery for diagnostic purposes.^23^

The reputation and experience of Jones’s fracture treatments and supporting those with permanent disabilities assisted him in securing influence and the necessary resources to plan his vision of an orthopaedic practice for treating disabled men during the First World War.^24^ Here, he applied his earlier experiences of treating people with disabilities through the principle of continuity and coordination of care through surgery and implementing a plan including physiotherapy, fresh air and purposeful activity.^25^ Notably, Jones directed and supervised the application of orthopaedic and rehabilitative facilities across Britain during the First World War. His involvement in the care of disabled ex-servicemen between 1914 and 1918 illustrates the contributions to Lancashire’s reputation as a pioneering medical institution and popular use of voluntary hospitals as civic philanthropy. This helped to gather political and financial support and provided an opportunity to engage in local social concerns on the treatments of the returning disabled ex-servicemen.

Upholding Lancashire’s reputation as a pioneering medical location, Manchester was one of the first towns to adopt a similar unit to Alder Hay with the addition of an orthopaedic unit at the Manchester Great Western Ducie Avenue building, Moss Side, with the surgeon and Captain of the RAMC (TF), Harry Platt as the surgeon in charge.^26^ Like Jones, Harry Platt was an acclaimed orthopaedic surgeon, gaining his medical training and capabilities at the Royal National Orthopaedic Hospital, London, and Ancoats Hospital in Manchester. During his position as surgeon to Ancoats Hospital, Platt pioneered Britain’s first specialist fracture clinic, another vital medical contribution used to treat and rehabilitate permanently disabled soldiers.^27^ Furthermore, Platt’s career as a leading surgeon is documented through an invaluable collection of papers examining the clinical experiments of orthopaedic injuries and investigations of the history of orthopaedic practice in Lancashire during the First World War.^28^ While many of the records within the papers remain closed, sources outlining the formation of orthopaedics at the Ducie Avenue and subsequent Grangethorpe House provide insight into the injuries of ex-servicemen returning to Manchester between 1914 and 1918 and the construction of an alliance between the region’s leading orthopaedic surgeons and neurologists and voluntary organisation administer an innovative scheme of care to support the reconstruction of the war-disabled men.

These medical centres allowed the region’s medical men to apply their clinical ideas within a substantial surgical infrastructure led by a network of medical colleagues specialising in Orthopaedics and Neurological treatments. Both facilities advocated the fundamental principles of providing a systematic care scheme and long-term rehabilitation. Equally, as Joanna Bourke and Roger Cooter have pointed out, this growth of orthopaedics during the First World War was not inevitable despite the demand. However, the opening of political space in medicine for this specialism was crucial in reorganising medical work that had been part of the modernist agenda since the 1880s.^29^ However, during the war, developments in the field gained support from Military Medical services, who stressed the need to invest in the aftercare available to ex-servicemen after hospital discharge. This led to national recognition for the construction of orthopaedic teams consisting of medical professionals and volunteers.

Over 200,000 wounded soldiers arrived in Manchester during the First World War, often after receiving unsuccessful care at other military hospitals. The Medical Case Sheets of the *Papers of Harry Platt* demonstrate how the rudimentary methods of treating orthopaedics, predominantly fractures, resulted in disproportionate numbers of men discharged from hospitals with permanent disabilities and no aftercare plan. Evidence would suggest that as orthopaedic treatments advanced, particularly within Manchester, servicemen were transferred to one of the six wards at the town’s Ducie Avenue unit. However, this increased Manchester’s casualties further, and orthopaedic care at Ducie Avenue was no longer sustainable.^30^

During a conference of East Lancashire’s mayors and chairmen at the Manchester Town Hall on 4 December 1916, Col. William Coates (Manchester’s RAMC (TF) commanding officer and chairman of the East Lancashire Joint Committee of the Order of St. John and British Red Cross Society) outlined his scheme to provide care for the ‘helpless men’.^31^ Referring to the work of the British Red Cross at the Star and Garter Home, he proclaimed that permanently disabled soldiers and those partially disabled were not sufficiently differentiated, and the accommodation provided for the former was inadequate across the country.^32^ Also, Coates deemed the construction of a home or homes in East Lancashire necessary and looked to the East Lancashire branch of the British Red Cross Society (BRCS) to enlist the support of influential Manchester Men who formed a committee, the East Lancashire Homes for Disabled Soldiers and Sailors.

The *Manchester Guardian* printed the committee’s proposal in December 1916, describing their intentions as ‘not looking to provide homes in the nature of convalescent but, to look after helpless men, to be waited on hand and foot, with the most careful nursing to make them comfortable’.^33^ Coates also considered the visitation of friends and relatives to homes and hospitals an essential part of regaining independence after disablement required to facilitate reintegration; therefore, the construction of local facilities was of great significance and relied on the support and goodwill of public charity.

Acknowledging the position of Manchester as a central disembarking point for returning soldiers during the First World War and the disregard for their welfare by the state, Coates enlisted the sympathies of Manchester’s philanthropic culture. Reinforced by the region’s humanitarian approach and commitment to constructing a systematic care system to support the challenges of war overlooked by the State, they advocated for specialised accommodation and rehabilitative centres to benefit the returning disabled ex-servicemen within East Lancashire. Subsequently, over £100,000 was raised to aid the purchase of suitable buildings for the permanently war-disabled and those registered under the War Charity Act (1916) as the East Lancashire Home for Disabled Sailors and Soldiers.^34^ Utilising the philanthropic sympathies of the region resulted in the purchase of four houses by the East Lancashire BRCS—Grangethorpe Hospital, Broughton House Salford (still in existence), Wyborne Gate Southport, and a private house in Blackburn to support the rehabilitation and recovery of ex-servicemen.^35^

Much of the initiative behind the development came from William Coates as a significant figure in organising medical care across the region and with a specific interest in improving the care and rehabilitation of disabled ex-servicemen, Coates frequently visited other military hospitals and Homes throughout Britain to remain apprised of the current care schemes available to support medical and social restoration.^36^ After visiting the Star and Garter Home for permanently disabled and paralysed soldiers and sailors in Richmond, Surrey, in 1916, Coates spoke with patients from the Northwest on their experiences at the home, which was intended to provide care for 60 men but was forced to accommodate over 200. The house was renowned for its extraordinary care and compassion towards disabled servicemen, many of whom were paralysed through either brain or spinal trauma or artillery injuries, resulting in total paralysis below the waist.^37^

Unlike military hospitals, the Star and Garter was a private home; therefore, it did not provide surgical treatments, focussing instead on retraining programmes and accommodation for those whose disabilities impeded the process of reintegration. In addition, with no systematic scheme of rehabilitation or provisions for permanently disabled servicemen outlined by the State in Britain before 1918, the establishment of the Star and Garter and its continued success relied on public charity, subscriptions and ambitious philanthropic efforts.^38^ While impressed by the work of the Star and Garter, Coates recognised that accommodation and long-term rehabilitation schemes for disabled men were inadequate.

## A Military Orthopaedic Hospital

**Fig. 1:**
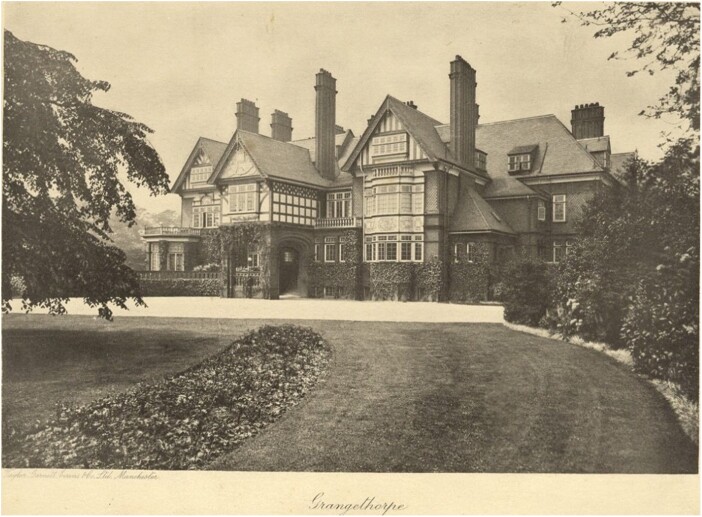
Grangethorpe Hospital, Rusholme 1917. A postcard showing the Grangethorpe Mansion House in 1917.^40^ Provided with permission by Chetham’s Library, Manchester and Rusholme Archives.

In 1917, due to an acute shortage of orthopaedic beds within Lancashire, the War Office prevailed upon William Coates and the East Lancashire BRCS to use Grangethorpe House to establish Manchester’s first orthopaedic hospital.^39^ Despite Coates’s initial proposal to transform the house into accommodation for permanently disabled soldiers, the British Red Cross handed Grangethorpe to the War Office for use as a military specialist orthopaedic centre, free of charge, supplying nursing staff the necessary equipment towards the general management of the hospital. Figure 1 shows the Grangethorpe Mansion House, set on 11 acres on the southern edge of Platt Fields, presenting an ideal solution to the shortage of orthopaedic facilities in the region. While Grangethorpe operated as a military orthopaedic hospital with the War Office undertaking complete control of the wards and surgical procedures, it granted the East Lancashire BRCS use of a section of the building to create ‘curative workshops’ for the discharged disabled ex-servicemen in East Lancashire requiring urgent rehabilitation.^41^

As the surgeon in charge of Manchester’s orthopaedics, Platt oversaw the conversion of Grangethorpe House. A report on Grangethorpe written by Platt in 1917 provides an in-depth description of the renovations, including an X-ray department, surgical theatres, a gymnasium and treatment rooms fitted with electrical therapy appliances and wax baths to aid rehabilitation.^42^ Also noted in Platt’s report was the significance of including curative workshops to ‘build a bridge between military and civilian life; to restore men and help make them useful citizens again’.^43^ The surgical work at Grangethorpe was overseen by Platt and John Stopford, an experienced military surgeon and neurologist, to Manchester’s 2nd Western General Hospital. Both men were interested in reconstructive surgery, predominantly peripheral nerve injuries, which characterised a substantial amount of the operations they performed and would later compile Platt’s publication in 1921, *The Surgery of the Peripheral Nerve Injuries of Warfare*.^44^

The Medical Case Sheets of Grangethorpe within the *Platt Papers* offer examples of orthopaedic practice within Lancashire during the First World War. The records available, primarily from 1918, evidence the medical journey and lack of aftercare that ex-servicemen experienced before their admission to Grangethorpe, and the prolonged symptoms orthopaedic injuries posed to their overall health. Individual cases describe the many injuries sustained by bullets and shrapnel, damaging limbs and the sciatic or ulnar nerve. Often diagnosed through symptoms of numb fingers and pain of the elbow, shoulder or wrist, most nerve injuries were treated with surgery by Stopford through nerve transportation surgery or ligament suturing followed by an aftercare plan of physical therapy, wax baths and massage therapy.^45^

A frequent injury noted within the *Platt Papers* was an un-united fracture of the humerus bone sustained by bullets. The demand for surgeons to perform treatments to severe fractures, delicate nerve transportation and supplying artificial limbs at Grangethorpe became overwhelming, mainly as the medical case sheets document previous unsuccessful treatments at other centres that needed correcting. These types of injuries were treated with an X-ray diagnosis, wound cleaning, surgery if necessary and then long-term rehabilitation, which became a recurrent clinical process in reconstructive treatment during the First World War. The treatments provided at Grangethorpe validated the advantages of restoring soldiers who would have otherwise died or been forced to live in disability and pain.^46^ Additionally, the methods and practices provided at Grangethorpe were comparable to Robert Jones’s work at the Shephard’s Bush Military Orthopaedic Hospital, and Grangethorpe would become more than just an orthopaedic hospital; it became a leading centre for rehabilitation specialising limb cases and nerve injuries.

## A Programme of Restoration and Rehabilitation

The supervision of care at Grangethorpe was overseen by several of Lancashire’s leading medical men. It characterised the region’s use of experimental treatments and forward-thinking therapies and the continuation of upholding its reputation as a society of medical pioneers. One example of this was Grangethorpe’s incorporation of rest and recuperation therapy into patients’ reconstruction plans, a significant medical development underpinned by the injuries of the First World War and widely used by doctors treating disabled servicemen requiring intense rehabilitation or prolonged periods of convalescence. Its use highlights the early signs of connecting war trauma and its effects on the serviceman’s mental health.

During the First World War, the idea of medical aftercare evolved further. It developed the foundations of modern-day occupational therapy, based primarily on recommendations of distinguished orthopaedic surgeon Robert Jones, whose reputation and experience in treating fractures and supporting those with permanent disabilities highlighted the benefits of prolonged aftercare in the social and medical rehabilitation of disabled people. Encouraged by his wealth of experience and leading medical knowledge, Jones outlined the inadequacies of the Government’s current policy, suggesting ‘a cohesion between departments of treatments, such as massage, physical therapies, which together make success in orthopaedic surgery.^47^ This idea was later manifested in the popular manual retraining schemes for disabled ex-servicemen between 1914 and 1918. These work-related therapies encouraged the disabled servicemen to re-engage with society and were offered collectively with physical therapies as part of the soldiers’ rehabilitative plan.

While the orthopaedic care of contemporary medicine follows a comparable system to Jones’s proposal, he was ahead of the time in his recommendations, particularly the need to administer a collaborative national hospital to treat the ailments of the war disabled. Nonetheless, his foresight generated significant changes in the acknowledgement of orthopaedic practice by the Government and shaped the foundations of future medical care available. Therefore, as a leading orthopaedic rehabilitative centre, Grangethorpe welcomed men from across Britain and contained innovative facilities and programmes following the guidance of Robert Jones and his scheme of prolonged and adaptable treatments to assist in rehabilitation.

Examples of rehabilitative facilities offered at Grangethorpe during the First World War are noted by a former member of the physiotherapy department who recalled diathermy, physiotherapy, sports and manual retraining workshops alongside occupational therapy and handicrafts.^48^ Also, like other orthopaedic centres, Grangethorpe provided printing presses and boot-making and repairing workshops to retrain the disabled men in curative and vocational skills vital for their return to civilian life and those men unable to return to previous employment.

Anna Carden Coyne described the unprecedented scale of the programmes needed for rehabilitation during the war period, with the premise of restoration underpinned by its pre-war history of work therapies and political ideology driven by usefulness and utilising occupational therapies to retrain men to a functional semi-skilled status.^49^ Indeed, the gendered approach to reconstruction was inadequate, focussing on returning *fit* soldiers to the front or military discharge to the care of voluntary organisations such as the British Red Cross, whose medical volunteers provided much of the retraining to prepare the disabled ex-servicemen for civilian life.^50^ However, the contribution of work therapies and manual retraining served as an essential form of healing, particularly in orthopaedic centres like Grangethorpe, where physical disabilities often overshadowed pride and masculine identities and were used in other comparable centres such as the Military Orthopaedic Hospital, Shephard’s Bush who drew extensively from work with disabled children.^51^

Grangethorpe’s incorporation of manual workshops as part of the rehabilitation scheme allowed the disabled ex-servicemen to exercise masculine traits and military discipline to overcome physical pain and give back to society whilst empowering them to escape the demands of soldiering and express their creativity.^52^ Drawing comparisons to the earlier workshops mentioned at the Henshaw’s Blind Society, where patients sold their handicrafts and furniture to Lancashire’s residents, work therapy shaped the public image of the disabled soldier with military hospitals and Red Cross facilities eager to show that men were not idle during recovery.^53^

Grangethorpe also provided a haven for disabled men to heal medically and socially through a scheme of medical volunteers and societies dedicated primarily to orthopaedic rehabilitation whilst contributing to the psychological support needed to reconstruct masculinity and the necessary independence to contribute to society. Examples of the range of rehabilitation therapies used by the doctors and medical volunteers of Grangethorpe are mentioned in the hospital’s Medical Services Records. These included weekly activities led by doctors in the gymnasium, equipped with rowing and cycle machines that promoted physical and mental strength, followed by time spent in the wax baths to improve blood circulation and reduce pain in injured joints and limb amputations.^54^

From the founding of the Manchester Royal Infirmary and the beginning of the region’s health system in 1752, Lancashire maintained its position as a leading medical institution through a probing network of prominent surgeons and doctors incorporating innovative and experimental treatments into effective remedies. This approach benefitted Grangethorpe’s rehabilitation unit considerably as its doctors’ expertise and commitment to medical science, notably those interested in physiotherapy, promoted the inclusion of ground-breaking medical equipment, including the use of the first Diathermy machine in England. With similar features to the wax baths, this surgical technique used a high-frequency electrical current to stimulate circulation and relieve pain by eliminating unhealthy tissue and alleviating joint stiffness, pain and muscle spasms, all frequent complaints of mutilated men.^55^

Photographs and personal testimonies presented by ex-patients and physiotherapy staff at Grangethorpe reinforce Lancashire’s philanthropic reputation and advanced medical institution through the centre’s insightful inclusion of sports therapies to enhance physical rehabilitation led by a 20-staff physiotherapy department.^56^ Throughout the First World War, the benefits of sports therapies gained national recognition, as doctors considered the outcomes similar to those achieved through other methods of physical treatments while also encouraging psychological healing.^57^ The implementation of athletics and competitive sports days, predominantly football, to stimulate physical and psychological recovery and advance healing was first promoted by renowned rehabilitative centres such as St. Dunstan’s and the blue Garter.^58^ While obtaining less awareness than other prominent rehabilitation centres, the sports and athletic therapies available at Grangethorpe were comparable to some of the leading rehabilitation centres in Britain. Crucially, sport was a routine part of military life, supporting the soldiers’ fitness and mental health during service and injury.

The structure and familiarity of sports during rehabilitation supplied disabled men with a space to begin restoring their masculinity away from the demands of military service and the anxieties of public life.^59^ Moreover, engagement in sports and athletics for servicemen readjusting to life with an amputation strengthened the proficiency of prosthesis during activities once considered routine, allowing a sense of normality and psychological release from the emasculation of disability. Also, as a team game, football embodied the spirit of comradeship and the male bonding experience of military service when men were forced to embrace group camaraderie to survive the horrors of warfare.^60^ During rehabilitation, disabled ex-servicemen explored new ways to reaffirm the spirit of comradeship and the sense of pride and mutual loyalty shared between pals as they continued to shape their lives as disabled civilians.

While Grangethorpe was under the command of the War Office, its rehabilitation unit relied on charitable contributions from the public and medical volunteers to sustain its prominent position. Recognising the vital role of public generosity alongside a growing curiosity in artificial limbs and disability, Grangethorpe invited spectators to the hospital’s disabled ex-servicemen’s charity sports days. While these events provided crucial physical fitness and social interaction for disabled patients alongside the opportunity to raise money towards their rehabilitation at Grangethorpe, most events relied on the organisation of volunteers and donations of sporting equipment by philanthropists.

**Fig. 2:**
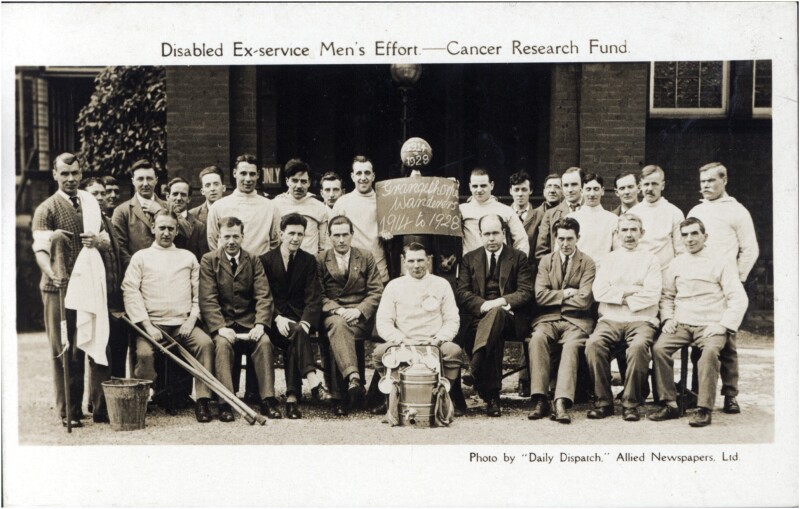
Grangethorpe Wanderers—Cancer Research Fund. A photo by ‘Daily Dispatch’, Allied Newspapers, Ltd showing the Grangethorpe Wanderers (1914–28) participating in a charity game of football entitled Disabled Ex-Service Men’s Efforts—Cancer Research Fund (date unknown).^61^ Provided with permission by Chetham’s Library, Manchester and Rusholme Archives

One example of the efforts of Grangethorpe’s war-disabled men was to raise funds and give back to society. Figure 2 illustrates the endeavours of the disabled ex-servicemen of the hospital’s team ‘Grangethorpe Wanderers 1914-1928’, suggesting patients participated in charitable football games to raise money for Cancer Research. While the palpable advantages and effectiveness of including sport and games therapies to physical and social rehabilitation were unquestionable, the provisions for their inclusion remained the responsibility of voluntary organisations and philanthropists, proving that little had changed within Lancashire’s public health and welfare during the First World War. Despite the advancements in medicine, ultimately, the responsibility of the disabled people and provisions of medical care in the absence of state welfare remained the duty of the region’s humanitarianism and philanthropic culture.

A final example of the rehabilitative programmes offered at Grangethorpe considers social reconstruction and expands on the region’s experimental medical heritage and incorporation of recreational therapies between 1914 and 1918. The restorative benefits of outdoor space and fresh air had been trialled on ‘mental’ patients of Manchester’s Asylum during the nineteenth century with a positive medical response. Patients were noted as exhibiting attributes of calmness and concentration after periods spent outdoors. The benefits of recreational therapy influenced the decisions of medical men and voluntary organisations of the First World War, who deemed the geographical location of any potential rehabilitative centres a priority. This medical opinion prompted the purchase of Grangethorpe House by the East Lancashire BRCS in 1916, described by William Coates in the Minutes of the Society as a ‘Quiet and idyllic location with surrounding gardens to furnish patients with a place of convalescence.’ The house would later provide the site for manual retraining workshops outlined by Robert Jones, who advocated for psychological healing by combining physical rehabilitation with outdoor respite.^62^

Historians have widely associated restoration in recreational space with the more severely disabled soldiers. For example, Julie Anderson, Peter Leese, Fiona Reid and Tracey Loughran equally focus on the benefits of the outdoors during rehabilitation schemes for those servicemen suffering from war neurosis and blindness due to sensory stimulation’s therapeutical features.^63^ Also, contemporary examples frequently feature photographs or films of blind ex-servicemen partaking in basket weaving and horticulture. However, the history of orthopaedic rehabilitation often overlooks the inclusion of social restoration, focussing instead on rigorous physical treatments and prosthetic limbs. Many therapies, including handicrafts, needlework and gardening, benefitted those with physical impairment and nerve damage, offering lighter exercise and aftercare, particularly for those recovering from delicate nerve operations. Furthermore, while these advancements demonstrated the success of alternative approaches to recovery, the responsibility of rehabilitation and the administration and trial of reconstructive therapies during the war relied on the efforts of medical voluntarists and charitable donations.

## Conclusion

This article examines the enormity of the restorative and medical challenges faced by the returning war disabled and those responsible for their care between 1914 and 1918. While the injuries of the First World War were unprecedented, the substantial number of disabled ex-servicemen returning to Britain requiring orthopaedic and neurological attention highlighted Britain’s pre-war inadequacies of social welfare and care of disabled people. Furthermore, Lancashire experienced a high proportion of orthopaedic injuries during this period, which challenged the region’s medical men and voluntary organisations, as well as the region’s medical heritage and experience in treating the physical disabilities of industrialisation. Therefore, the construction of Grangethorpe Hospital in 1917 was instrumental in providing innovative schemes of rehabilitation to disabled ex-servicemen within Lancashire.

The support offered through the shared work of pioneering orthopaedic doctors, physicians and organised charities that gained public support and financial backing was instrumental in advocating the advantages of private facilities providing specialised orthopaedic treatments. Furthermore, the dedicated role of Grangethorpe’s medical volunteers embodied the region’s charitable reputation and concentrated on preparing disabled ex-servicemen, both physically and psychologically, with the skills to gain employment and contribute to society. Finally, the investigation of care provisions provided at Grangethorpe Hospital between 1914 and 1918 exhibits the freethinking attitudes of Lancashire’s volunteers, who created a culture representative of a shifting attitude towards disability and inequality. This hospital demonstrates the region’s status as a pioneering medical facility efficient in providing innovative schemes of rehabilitation to support the social and physical reconstruction of disabled ex-servicemen during the First World War.

## Footnotes

1 Peter Grant, *Philanthropy and Voluntary Action in the First World War: Mobilizing Charity* (New York: Routledge, 2014), 24–7.

2 Hugh Cunningham, *The Reputation of Philanthropy Since 1750: Britain and Beyond* (Manchester: Manchester University Press, 2020), 169; Emily Bartlett, ‘Reassembling Disabled Identities: Employment, Ex-servicemen, and the Poppy Factory’, *Journal of Social History*, 2019, 54, 212.

3 Deborah Cohen, *The War Come Home: Disabled Veterans in Britain and Germany*, *1914-1939* (London: University of California Press Ltd, 2001), 19.

4 Ibid., 8.

5 Meaghan Kowalsky, ‘“This Honourable Obligation”: The King’s National Roll Scheme for Disabled Ex-Servicemen 1915–1944’, *European Review of History*, 2007, 14, 567–72.

6 Joanna Bourke, *Dismembering*, 32–3.

7 Bourke, *Dismembering*, 34.

8 Ibid., 33–40.

9 Ibid., 51.

10 Ibid., 568.

11 Jeffrey S. Reznick, *Healing the Nation*: *Soldiers and the Culture of Caregiving in Britain During the Great War* (Manchester: Manchester University Press, 2004), 120–3.

12 Stella V. F. Butler, ‘Academic Medicine in Manchester: The Careers of Geoffrey Jefferson, Harry Platt and John Stopford 1914-39’, *Bulletin of John Rylands Library*, 2005, 87, 7.

13 B. Di Matteo, V. Tarabella, G. Filardo, *‘*Sir Robert Jones: Orthopaedic Surgeon and War Hero’, *International Orthopaedics*, 2015, 39, 1021–5.

14 Robert Jones, *Notes on Military Orthopaedics* (London: Cassell, 1917), 60.

15 Manchester Ship Canal, *University of Manchester Library* GB 133 PWR/4/1.

16 Frederick Watson, *The Life of Sir Robert Jones* (London: W. Wood & Company, 1934), 98–103.

17 Ibid.

18 A. J. Carter, ‘Heroic Medicine’, *The British Medical Journal*, 1991, 303, 21–8.

19 G. R. Girdlestone, ‘The Robert Jones Tradition’, *The Journal of Bone and Joint Surgery*, 1947, 1–9.

20 Ibid., 8.

21 A. J. Carter, ‘Heroic Medicine’, 21.

22 Agnes Hunt, *This Is My Life* (London: Blackie & Sons Ltd, 1934), 75–80.

23 Ibid., 75–7.

24 Butler, ‘Academic Medicine’, 7.

25 Ibid., 139.

26 Patricia Gray, ‘Grangethorpe Hospital Rusholme 1917-1929’, *Transactions of the Lancashire and Cheshire Antiquarian Society*, 1975, 78, 52–5.

27 ‘Records of Orthopaedic Practice’, *Papers of Sir Harry Platt 1904-1986*, The University of Manchester Library, GB 133 PLA/1.

28 Ibid.

29 Roger Cooter, *Surgery and Society in Peace and War: Organisation of Medicine*, *1880-1948*, 46–50; Joanna Bourke, *An Intimate History of Killing* (Great Britain: Granta Books, 1999), 108.

30 John V. Pickstone, *Medicine*, *and Industrial Society: A History of Hospital Development in Manchester and Its Region 1752-1946* (Manchester: Manchester University Press, 1985), 207.

31 Alfred Keogh, ‘The Medical Service for The Proposed Territorial Force’, *Lancet*, 1907, 2.

32 Anon, ‘Home for Disabled Soldiers’, *Manchester Guardian*, 6 December 1916.

33 Ibid.

34 GB 133 MMC/8/30 British Red Cross Society, 1914-1970, *Manchester University Library.*

35 Patricia Gray, ‘Grangethorpe Hospital’, 1975, 78, 54.

36 Julie Anderson, *Soul of a Nation War Disability and Rehabilitation in Britain* (Manchester: Manchester University Press, 2011), 43.

37 Ibid., 44.

38 Deborah Cohen, *The War Come Home*, 17.

39 Anon, ‘For Disabled Soldiers’ and Sailors’, *Manchester Guardian*, 22 September 1917.

40 Postcard showing Grangethorpe Hospital’s main house dated 1917. With permission from Chetham’s Library and Rusholmearchives.org.

41 Anon, ‘Orthopaedic Surgery: New Manchester Hospital’, *Manchester Guardian*, 16 October 1916.

42 ‘Grangethorpe Hospital 1917-1926’, *Papers of Sir Harry Platt*, University of Manchester Library, GB 133 PLA/1/2.

43 Ibid.

44 Harry Platt, ‘The Surgery of the Peripheral Nerve Injuries of Warfare’, *British Medical Journal*, 1921, 1, 596–600.

45 Grangethorpe Hospital, *Sir Harry Platt*, GB 133 PLA/1/2.

46 Pickstone, *Medicine and Industrial Society*, 207.

47 Quoted in Frederick Watson, *The Life of Sir Robert Jones* (London: W. Wood & Company, 1934), 164–5.

48 Patricia Gray, *Grangethorpe Hospital*, 55–9.

49 Anna Carden Coyne, ‘Butterfly Touch: Rehabilitation, Nature and Haptic Arts of the First World War’, *Critical Military Studies*, 2016, 6, 2–3.

50 Seth Coven, ‘Remembering and Dismemberment: Crippled Children, Wounded Soldiers and the Great War in Britain,’ *The American Historical Review*, 1994, 4, 1167–202; Reznick, *Healing the Nation*, 120.

51 Ibid., 119.

52 Anna Carden Coyne, *Butterfly Touch*, 177.

53 Ibid., 187.

54 Medical Services, *University of Manchester Library*, GB 133 MMC/9/49/2.

55 Patricia Gray, *Grangethorpe Hospital*, 55.

56 Grangethorpe Hospital Photographs, *Platt Papers University of Manchester Library* GB 133 PLA/1/21917-1926 and papers of Sir Harry Platt, *Grangethorpe Hospital Patient Case Sheets* GB 133 PLA/1/2/2.

57 Patricia Gray, 58.

58 Julie Anderson, *Healing the Nation*, 40–3.

59 Anna Carden Coyne, ‘Masculinity and the Wounds of the First World War: A Centenary Reflection’, *French Journal of British Studies*, 2015, 1–7.

60 Ibid., 7.

61
Figure 2: Postcard showing ‘Grangethorpe Wanderers’ (1914–1928), Disabled ex-servicemen and staff members participating in a charitable Cancer Research Fund. With permission from Chetham’s Library, Manchester, and Rusholme Archives.

62 William Coates, *East Lancashire Branch British Red Cross Society: An Illustrated Account of the Work of the Branch During the First Year of the War* (Sherratt and Hughes, 1916).

63 For an overview of the history on rehabilitation for the blind and shell shocked Soldiers and Sailors, see: Julie Anderson, *War Disability and Rehabilitation in Britain* (Manchester: Manchester University Press, 2011); Tracey Loughran, *Shell-Shock and Medical Culture in First World War Britain* (Cambridge: Cambridge University Press, 2017); Fiona Reid, *Broken Men: Shell Shock, Treatment and Recovery in Britain 1914-30: Shell Shock, Treatment and Recovery in Britain 1914-1930* (London: Bloomsbury Academic, 2011); Peter Leese, *Shell Shock: Traumatic Neurosis and the British Soldiers of the First World War* (Hampshire: Palgrave Macmillan, 2002).

## Notes

PhD candidate in History at Manchester Metropolitan University. Received a BA (Hons) in Social History & History (2019) and MRes History (2021) from Manchester Metropolitan University. PhD Thesis is entitled ‘The Continuity of Poor Law Policy: A Hierarchy of *Undeserving* and *Deserving* First World War Disabled Ex-Servicemen in Lancashire.’ My research explores the continuity of the Poor Law classifications of deserving and undeserving and its influence on social policies for disabled ex-servicemen with mental health (dis)orders and sight loss within Lancashire.

Chetham’s Library and rusholmearchive.org have provided permission to include two images of Grangethorpe Hospital, Rusholme.

